# Adaptations in clinical examinations of medical students in response to the COVID-19 pandemic: a systematic review

**DOI:** 10.1186/s12909-022-03662-7

**Published:** 2022-08-05

**Authors:** Sapphire Cartledge, Derek Ward, Rebecca Stack, Emily Terry

**Affiliations:** University of Birmingham, Birmingham, B15 2TT UK

**Keywords:** Undergraduate medical education, COVID-19, Clinical examination, OSCE

## Abstract

**Introduction:**

Clinical examinations (assessments) are integral to ensuring that medical students can treat patients safely and effectively. The COVID-19 pandemic disrupted traditional formats of clinical examinations. This prompted Medical Schools to adapt their approaches to conducting these examinations to make them suitable for delivery in the pandemic. This systematic review aims to identify the approaches that Medical Schools, internationally, adopted in adapting their clinical examinations of medical students in response to the COVID-19 pandemic.

**Methods:**

Three databases and four key medical education journals were systematically searched up to 22 October 2021; a grey literature search was also undertaken. Two reviewers independently screened at title, abstract stage and full text stage against predefined eligibility criteria. Discrepancies were resolved by discussion and involvement of senior authors. Risk of bias assessment was performed using an adapted version of a pre-existing risk of bias assessment tool for medical education developments. Results were summarised in a narrative synthesis.

**Results:**

A total of 36 studies were included, which documented the approaches of 48 Medical Schools in 17 countries. Approaches were categorised into in-person clinical examinations (22 studies) or online clinical examinations (14 studies). Authors of studies reporting in-person clinical examinations described deploying enhanced infection control measures along with modified patient participation. Authors of studies reporting online clinical examinations described using online software to create online examination circuits. All authors reported that adapted examinations were feasible, scores were comparable to previous years’ student cohorts, and participant feedback was positive. Risk of bias assessment highlighted heterogeneity in reporting of the clinical examinations.

**Conclusions:**

This review identified two broad approaches to adapting clinical examinations in the pandemic: in-person and online. Authors reported it was feasible to conduct clinical examinations in the pandemic where medical educators are given sufficient time and resources to carefully plan and introduce suitable adaptations. However, the risk of bias assessment identified few studies with high reporting quality, which highlights the need for a common framework for reporting of medical education developments to enhance reproducibility across wider contexts. Our review provides medical educators with the opportunity to reflect on past practises and facilitate the design and planning of future examinations.

## Background

Clinical examinations, or assessments, are integral to ensuring medical students are competent to progress to higher levels of training or a medical qualification [1, 2]. The most widely used form of clinical examination is the Objective Structured Clinical Examination (OSCE) [3–5]. OSCEs involve candidates rotating around a circuit of stations where each station has a different task ranging from procedural skills to history taking.

The COVID-19 pandemic and subsequent implementation of social distancing rules disrupted traditional formats of clinical examinations [6, 7]. Typically, these examinations involve numerous participants, including candidates, examiners and patients, performing tasks such as physical examinations in a confined venue. These formats were no longer appropriate for delivery in the early months of the COVID-19 pandemic, therefore Medical Schools had to adapt swiftly their approaches to clinical examinations in order to conform with local and national COVID-19 restrictions, and to ensure the safety of all participants [8, 9].

Three systematic reviews have investigated medical education developments due to the pandemic [10–12] of which two considered developments in assessment [11, 12]. Gordon et al. [12] subsequently conducted an updating scoping review [13] and identified a growing body of literature on adaptations to clinical examinations due to the COVID-19 pandemic, concluding that there was now a need for a systematic review focussed on assessment to capture and summarise developments in this field. Medical educators now need an up-to-date review of policy and practice changes instituted in order to learn from the experiences of the last two years, inform future designs for clinical examinations, and determine what could and should remain post-pandemic to facilitate efficient, safe and effective assessment practice. We aimed to address this need by undertaking a systematic review that addressed the following three main questions:

- How were clinical examinations adapted in response to the COVID-19 pandemic? (i.e., description or ‘what was done?’)
- What were the successes and challenges of designing and implementing clinical examinations? (i.e., evaluation or ‘what went well and what didn’t?’)
- What are the recommendations for future practices informed by lessons learnt by the study authors? (i.e., implications or ‘what’s next?’)

## Methods

Our systematic review was conducted from January to October 2021. Prior to commencing, a study protocol was uploaded onto the Center for Open Science (OSF) registry (10.17605/OSF.IO/R64NZ) [14]. We conducted this review in accordance with the STructured apprOach to the Reporting In healthcare education of Evidence Synthesis (STORIES) statement [15] and the Best Evidence Medical Education (BEME) review guidance [16].

### Search strategy

Our original search took place in February 2021. However, because of the topical nature of this review and the rate at which new literature is emerging, we conducted a final updating search on 22^nd^ October 2021 using the same search strategy as described below.

We searched three electronic databases: MEDLINE, EMBASE and ERIC (Education Resources Information Centre). We piloted our search strategy on MEDLINE. Our final search strategy consisted of three axes which were combined with Boolean operators: (one representing the COVID-19 pandemic) AND (one representing medical education) AND (one representing clinical examination). Each axis consisted of keywords with their truncated forms and Medical Subject Headings (MeSH)/subject headings or descriptors specific to the database. We cross-checked our database searches against our searches of indexed journals to confirm the database searches captured papers identified from our journal searches.

In addition to our database search, we hand-searched the online publications of four key medical education journals (The Clinical Teacher, Medical Education, Medical Teacher and MedEdPublish) using the same Boolean combination of axes described above.

We also included Google Scholar in our search as it captured international texts and other forms of non-peer reviewed material. Since Google Scholar yields large volumes of irrelevant results, we made the decision prior to the search to stop screening when no results were passing through title and abstract screening after two pages of results (20 hits) [17].

We searched for grey literature on the An International Association for Medical Education (AMEE) website [18], including the COVID-19 page with webinars [19] and the virtual conference book from September 2020 [20]; The Association for the Study of Medical Education (ASME) website [21], and ASME-Bite-Size Youtube playlist [22] (a platform created for ASME members and the wider medical education community to discuss challenges faced in the COVID-19 pandemic and disseminate good practices) [21, 22]; and the MedEdPORTAL website [23], including the virtual learning resources during COVID-19 page [24].

Results were exported onto Endnote reference manager [25] and subsequently to Rayyan systematic review software [26].

### Study eligibility

The SPIDER model [27], a search strategy tool used for qualitative research, was used to refine our review question and determine study eligibility criteria.

We defined a structured clinical examination as an examination in a simulated clinical environment in which candidates perform pre-designed tasks, are examined by appointed examiners, and where multiple candidates are examined in turn. This contrasts with a work-based assessment which we define as individual or small groups of students being assessed on placement in a hospital or clinical environment by a clinician.

Inclusion and exclusion criteria were piloted at the title and abstract screening stage (first 100 results) and full text screening stage (first 10 results).

The following inclusion criteria were applied:

- Studies describing how one or more Medical Schools adapted their clinical examination of medical students in response to the COVID-19 pandemic
- Studies describing adaptations to any type of non-work based, structured clinical examination (e.g., OSCE)
- Studies including medical students in any year of study
- Studies available as pre-publications and non-peer reviewed material in addition to published articles in peer reviewed scientific journals
- Studies from any location and in any language

The following exclusion criteria were applied:

- Studies describing any non-clinical, knowledge-based examinations (e.g., recall written examinations)
- Work based assessments
- Studies including examination candidates with any form of provisional medical registration
- Opinion pieces where authors do not include a description of the adaptations to the design and implementation of their own deployed clinical examination

Two independent reviewers (SC and ET) screened titles, abstracts and full text articles against the inclusion and exclusion criteria. Rayyan [26] was used to record independent screening decisions by the two reviewers and conflicts were discussed with the additional involvement of senior authors if agreement was not reached. Inter-rater reliability was calculated at each screening stage; prior to commencing we set a threshold value for Cohen’s Kappa of 0.61 and if it was below this value, a senior reviewer would also conduct the screening and compare with the other two reviewers’ results. Results were reported according to ‘Preferred Reporting Items for Systematic Reviews and Meta-Analyses’ (PRISMA) statement for referred reporting items for systematic reviews and meta-analyses [28].

### Data extraction

We used Excel to create a data extraction form. Initially, one reviewer (SC) piloted the data extraction form on five studies. Two independent reviewers (SC and ET) performed data extraction on a random sample of 15% of the included studies to check for alignment then subsequently SC performed data extraction on the other 85%. Broadly, data were extracted in three categories: study characteristics, what was reportedly done and study author evaluations.

### Risk of bias

Currently, there is no consensus method for assessing study quality in studies included in medical education systematic reviews [12, 29–31]. Authors’ postulate this is due to the complex nature of medical education developments [12]. Therefore, we adapted a pre-existing tool [12] previously used to assess reporting bias in this context [31–33].

Our final version of the tool rated underpinning bias, setting bias, resource bias and evaluation bias as high quality, unclear quality, or low quality (Table 1). This tool appraises how study authors reported the implementation and conduct of the revised clinical examination, so while it is an indicator of study quality, it does not directly assess the quality of the intervention itself; it relies on the subjective judgement of the reviewer due to the absence of marked quantitative thresholds. Therefore, we use the term ‘risk’ of bias, and we included all papers in the narrative synthesis with no respective weightings given to studies due to their quality score.

**Table 1:** Risk of bias assessment (adapted from Gordon et al. [12])

Bias source	High quality	Unclear quality	Low quality
Underpinning bias	Clear description of the reasoning underpinning adaptations to the clinical examination including local COVID-19 restrictions	Some limited discussion of underpinning, with no description of local COVID-19 restrictions	No mention of underpinning
Setting bias	Clear details of the assessment location and participant characteristics (of examiners, candidates and patients) in the study	Some limited description of setting and participant characteristics	No details of participant characteristics or setting
Resource bias	Clear description of the cost / time / resources needed for the clinical examination	Some limited description of resources	No mention of resources
Evaluation bias	Detailed evaluation of the clinical examination in the form of reflections or quantitative evaluation with explanation	Some limited evaluation of the clinical examination	No evaluation

Two reviewers (SC and ET) performed a risk of bias assessment on a random sample of 15% of the studies to check for alignment and identify major discrepancies followed by SC assessing the remaining 85%. Again, inter-rater reliability was calculated using Cohen’s Kappa, with the same threshold as above.

### Narrative synthesis

Because of the descriptive nature of the studies, we planned to summarise results in a narrative synthesis using guidance from Popay et al. [34]. This approach recommends using tables to identify common practises in the phenomena of interest and group studies accordingly. This led to grouping studies into ‘online examinations’ or ‘in-person examinations’. Further sub-categorisation was also determined using the same approach utilising the data extraction form headings. Our narrative synthesis broadly aimed to describe ‘what was done’ and ‘study author reflections’ in line with our review’s aims, supplemented with the use of visual aids such as photos or tables to report the general characteristics of studies, as well as risk of bias score.

## Results

### Study selection

**Fig. 1:**
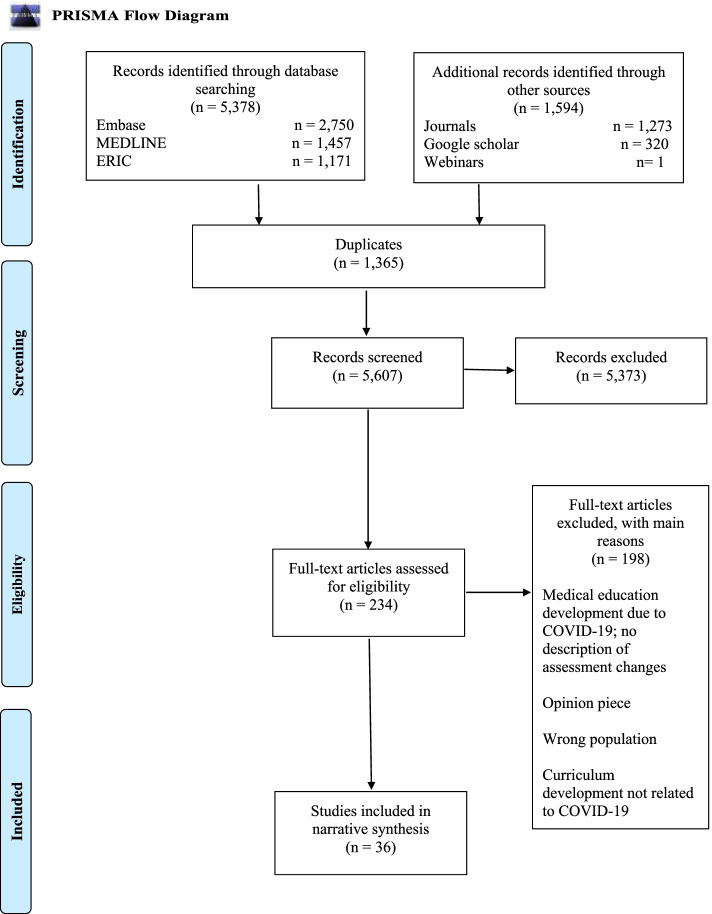
PRISMA flow diagram [28]

Our search strategy identified a total of 6,972 hits, which following de-duplication, resulted in 5,628 unique records (Fig. 1). Following screening at title and abstract stage, 255 articles were obtained for screening at the full text stage. The primary reasons for exclusion at full text stage were: opinion pieces in which the authors were not reporting direct experiences of adapting or delivering an examination; studies describing medical education initiatives, but not specifically clinical examinations; studies describing other types of examination (e.g. theory based); and examinations in the wrong population (not medical students). Subsequently, 36 studies were included in the narrative synthesis.

Considering the two reviewers, Cohen’s Kappa was 0.79 at title and abstract stage and 0.92 at full text stage, representing substantial agreement.

### Study characteristics

#### General study characteristics

Some studies reported findings from multiple Medical Schools, and some Medical Schools published multiple studies, therefore the 36 included studies reported the approach adopted by 48 Medical Schools. Twenty-two studies were published in 2020 and fourteen in 2021 with an overall date range from March 2020 to September 2021. Studies reported findings from 17 countries in six continents. The largest number of studies came from Asia (39%) and Europe (36%). One study required translation from Spanish into English using an externally sourced translator before inclusion.

Twenty-seven studies primarily focused on structured clinical examinations while 9 described clinical examinations as part of a wider focus on medical education developments in the pandemic as a whole.

#### Types of clinical examinations

Thirty-four studies described adaptations to OSCEs, one described adaptation to the M3 structured clinical examination (the German state licensing examination taken after six years of Medical School), and one described development of the Virtual Clinical Encounter Examination (VICEE; a structured examination designed to predominantly assess non-psychomotor clinical skills). Of the thirty-four studies describing OSCEs, thirteen described end of year OSCEs; eleven described end of rotation/clerkship OSCEs; five described supplementary OSCEs; and five did not specify the type of OSCE examinations.

#### Reported outcomes

Out of the 36 studies, only ten reported quantitative outcomes comparing candidate scores from previous years to the present year, and 24 reported either formal (e.g., survey) or informal feedback from stakeholders and participants who took part in the clinical examinations. Furthermore, six studies reported the number of COVID-19 cases in participants following in-person clinical examinations.

### Risk of bias

Only three studies scored highly in all four domains in the risk of bias assessment tool (Table 2) [35–37]. Two of these studies were by the same author group [35, 36], and the description of the examinations by all three studies were thorough enough to allow replication. No studies scored low quality in all four domains; the two lowest rated studies scored low quality in two domains and unclear quality in two domains [38, 39]. Of these, one study was a letter to the editor, meaning it did not undergo the same rigorous review as a research paper [38]; and the other study did not focus on clinical examinations as its primary aim, rather it was a description of adaptations to medical education as a whole [40].

**Table 2:** Study characteristics and risk of bias assessment

**Author(s)**	**Publication date**	**Type of study**	**Geographical location**	**Medical School**	**Type of clinical examination**	**In-person or online examination**	**Number of candidates; stations**	**P**^a^	**S**	**R**	**E**
Adeleke et al. [41]	1.12.20	Published case report	South Africa	Walter Sisulu University	6^th^ year family and rural health OSCE	In-person	-;-	H	U	U	L
Ashokka et al. [42, 43]	13.5.20 27.5.20	Published case report Webinar	Singapore	National University Health System	OSCE	In-person	-;-	H	L	U	L
Bastanhagh et al. [38]	17.11.20	Published case report	Iran	Tehran University of Medical Sciences	OSCE	In-person	-;-	U	L	L	U
Bauer et al. [44]	3.12.20	Published case report	Switzerland	University of Bern	3^rd^ year OSCE	In-person	-;-	H	H	U	H
Boursicot et al. [35]	27.3.20	Published case report	Singapore	Duke-National University Singapore Medical School	Final year (graduate medicine) OSCE	In-person	-;-	H	H	H	H
Canning et al. [36]	19.8.20	Published case report	Singapore	Duke-National University Singapore Medical School	Supplementary final year (graduate medicine) OSCE	In-person	-;-	H	H	H	H
Fritsche et al. [45]	3.12.20	Pre-print case report	Germany	Martin-Luther-Universität Halle-Wittenburg, Medizinische Fasultät	6^th^ year M3 structured examination (German state licencing examination)	In-person	-;-	U	H	U	U
Lee et al. [46]	2.2.21	Published commentary case report	Hong Kong	The University of Hong Kong, department of medicine	Final year OSCE	In-person	-;-	H	H	U	H
Lee et al. [47]	16.7.20	Pre-print case report	Hong Kong	Li Ka Shing Faculty of Medicine, The University of Honk Kong	Final year OSCE	In-person	-;-	H	U	U	U
Lengerke et al. [48]	3.12.20	Published case report	Germany	Hannover Medical School	2^nd^ year OSCE	In-person	-; 8	H	U	U	U
Ngiam et al. [49]	18.10.20	Published short report	Singapore	Yoo Loo Lin School of Medicine, National University of Singapore	Final year OSCE	In-person	-;-	H	U	U	U
Nourkami-Tutdibi et al. [50]	28.1.21	Published short report	Germany	Saarland University	Ultrasound and echocardiography course OSCE	In-person	45 in abdominal ultrasound group and 30 in echocardiography; -	H	U	U	U
Samaraseke et al. [40]	6.5.20	Published case report	Singapore	Yoo Loo Lin School of Medicine, National University of Singapore	End of year OSCE	In-person	-;-	H	U	U	U
Wiedenman et al. [39]	6.4.21	Published case report	Germany	Universitätsklinikum Freiburg	Ophthalmology end of rotation OSCE	In-person	164; 3	U	L	L	U
Anraham et al. [51]	9.3.21	Published short report	Canada	University of Alberta	2^nd^ year OSCE	Online	-; -	L	H	U	H
Blythe et al. [52]	20.4.21	Published case report	UK	Barts and the London School of Medicine and Dentistry	Final year supplementary OSCEs	Online	9; 5	L	H	U	H
Boyle et al. [53]	18.12.20	Published case report	UK	School of Medicine, Nursing and Dentistry Glasgow	High stakes supplementary examinations OSCE	Online	-; -	L	H	U	H
Brown et al. [54]	11.8.21	Poster	UK	Bristol University	Psychiatry mock OSCE	Online	-;-	U	H	U	U
Conti et al. [55]	11.8.21	Poster	UK	Queens University Belfast	Final year psychiatry mock OSCE	Online	24; 4	U	U	U	U
Craig et al. [56]	5.7.20	Published case report	Canada	University of British Columbia	4^th^ year supplementary OSCE	Online	4; 7	U	U	H	H
Faria et al. [57]	7.5.20	Published case report	Brazil	Centro Universitario Christus- Campus Parque Ecologico	3^rd^ year OSCE	Online	-; 3	U	H	H	H
Farrell et al. [58]	17.5.21	Abstract pre-print	USA	Harvard Medical School	OSCE	Online	160; 2	U	H	H	H
Gracía-Seoane et al. [59]	5.2.21	Pre-print case report	Spain	16 Spanish participating Medical Schools	Final year OSCE	Online	2829; 10	U	H	U	H
Hamdy et al. [60]	15.06.21	Published case report	UAE	Gulf Medical School	Final year VICEE	Online	61; 5	U	H	U	H
Hannon et al. [61]	16.5.20	Published case report	USA	School of Medicine, University of Utah	Surgery, medicine and neurology clerkship OSCEs	Online	49; 2	U	H	H	H
Hopwood et al. [62]	20.10.20	Published case report	UK	University College London Medical School	OSCE	Online	-; 18	U	H	H	H
Lara et al. [63]	20.8.20	Published case report	USA	Uniformed Services University Betheseda	Paediatric clerkship OSCE	Online	49; 4	U	H	H	U
Major et al. [64]	24.4.20	Published in practise report	Qatar	Weill Cornell Medical School	3^rd^ year women’s reproductive and sexual health OSCE	Online	9; -	U	H	H	H
Martinez et al. [65]	24.8.20	Published case report	USA	Florida Atlantic University Charles E. Schmidt College of Medicine	1^st^ year OSCE	Online	47; -	U	L	L	H
Ryan et al. [66]	30.9.20	Published case report	Australia	University of Melbourne Doctor of Medicine program	2^nd^ year (graduate medical students) OSCE	Online	361; 2	U	H	H	H
Setiawan et al. [67]	27.7.21	Published case report	Indonesia	Sultan Agung Islamic University	4^th^ and 5^th^ year surgical OSCE	Online	270; -	U	U	U	L
Shaban et al. [68]	26.09.21	Published case report	UAE	United Arab Emirates University	3^rd^, 4^th^ and 6^th^ year end of year OSCEs	Online	3^rd^ year: 80,10 4^th^ year: 69,9 6^th^ year: 73,10	U	U	U	L
Shaiba et al. [69]	26.07.2021	Published case report	Saudi Arabia	College of Medicine, King Saud University	Paediatric OSCE for final year students	Online	-;-	U	U	U	L
Shehata et al. [70]	16.10.20	Published case report	Kingdom of Bahrain	College of Medicine and Medical Sciences, Arabian Gulf University	OSCE	Online	3; 8	U	U	H	H
Shorbagi et al. [37]	27.7.21	Pre-print case report	UAE	University of Sharjah	End of clerkship OSCE	Online	105–108; -	H	H	H	H
Stewart et al. [71]	14.1.21	Published case report	UK	University of Buckingham	Final year supplementary OSCE	Online	-; -	U	H	U	H

^a^P = underpinning bias, S = setting bias, R = resource bias, E = evaluation bias

H = high quality; U = unclear quality; L = low quality

### Summary of the clinical examinations

Studies reported either in-person or online clinical examinations amended or newly developed in response to the pandemic; fourteen studies described in-person examinations and 22 described online examinations (Table 2). We assumed that when authors described standardised or simulated patients, they were referring to an individual trained to act as a patient. However, we have used the same descriptive terms that the authors used in their studies in our reporting of results.

### In-person examinations

#### What was done?

##### Infection control measures

**Fig. 2:**
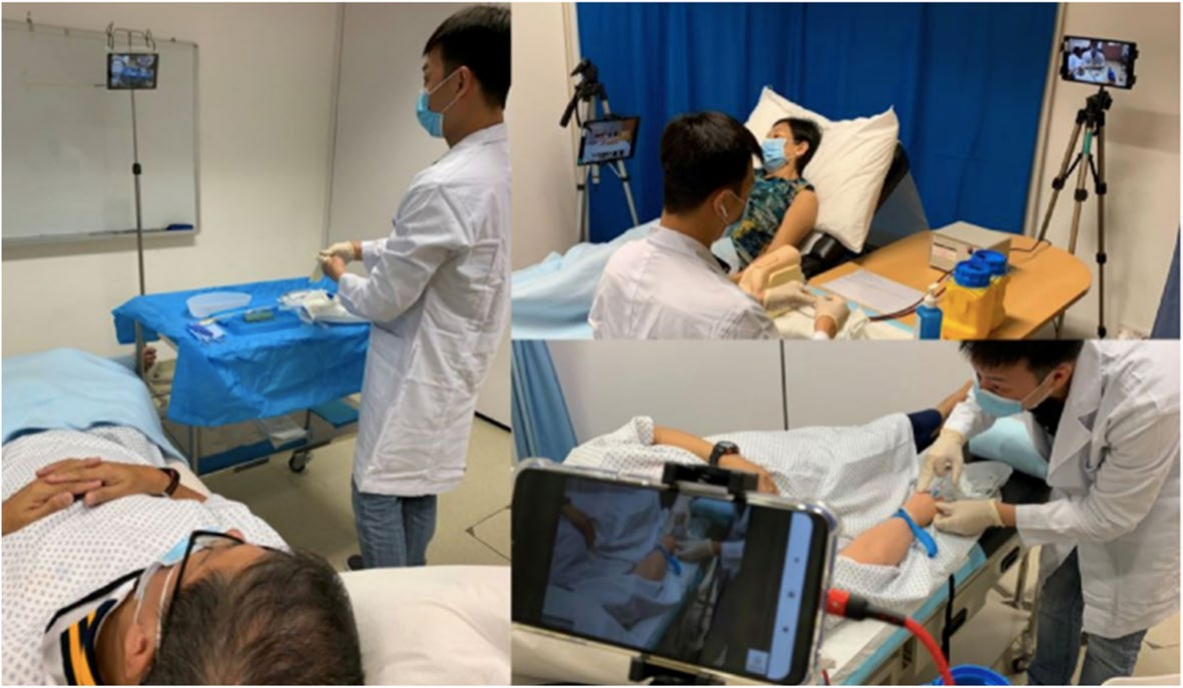
An image from an OSCE with infection control measures (off-site examiner using video-conferencing; PPE) at Duke-NUS Medical School [72]. (Taken with permission from the study author. Participants and simulated patients in the photo gave written consent.)

All studies describing in-person examinations described enhanced infection control measures deployed to minimise risk of transmission of COVID-19 (Table 3). These measures included reduced participant numbers, the use of personal protective equipment (PPE) by all participants and even remote examiners using videoconferencing systems to observe stations (Fig. 2, a visual representation of several infection control measures at Duke-NUS University, Singapore).

**Table 3:** Infection control measures described by study authors

Infection control measures:	Studies:
Temperature screening prior to entry	Bastanhagh et al. [38] Ngiam et al. [49]; Canning et al. [36]; Fritsche et al. [45]; Lee et al. [46]; Lee et al. [47]; Samaraseke et al. [40]
Health declaration forms (travel history, contact tracing)	Bastanhagh et al. [38]; Boursicot et al. [35]; Canning et al. [36]; Ngiam et al. [49]
PCR testing two days beforehand	Lee et al. [46]
Examination held in non-clinical institute	Bastanhagh et al. [38]; Bauer et al. [44]; Boursicot et al. [35]; Canning et al. [36]; Fritsche et al. [45]; Lee et al. [46]; Lee et al. [47]; Lengerke et al. [48]; Samaraseke et al. [40]
Social distancing	Bastanhagh et al. [38]; Bauer et al. [44]; Boursicot et al. [35]; Canning et al. [36]; Fritsche et al. [45]; Lee et al. [46]
Masks	Bastanhagh et al. [38]; Ngiam et al. [48]; Bauer et al. [44]; Boursicot et al. [35]; Canning et al. [36]; Lee et al. [46]; Lengerke et al. [48]; Nourkami-Tutdibi et al. [50]; Samaraseke et al. [40];
Hand hygiene	Canning et al. [36]; Lee et al. [46]; Nourkami-Tutdibi et al. [50]; Samaraseke et al. [40]
Equipment disinfectant	Bastanhagh et al. [38]; Ngiam et al. [49]; Bauer et al. [44]; Canning et al. [36]; Lee et al. [46]; Nourkami-Tutdibi et al. [50]
Using reusable bedding gowns	Boursicot et al. [35]; Lee et al. [46]
Reduced number of participants onsite	Ashokka et al. [42, 43]; Bastanhagh et al. [38]; Boursicot et al. [35]; Canning et al. [36]; Fritsche et al. [45]
Cohorting (staff from one clinical institute participate in one OSCE circuit separate to other circuits created for other clinical institutes)	Ashokka et al. [42, 43]; Boursicot et al. [35]; Ngiam et al. [49]; Samaraseke et al. [40]
Examiners examined remotely using videoconferencing software (Fig. 2)	Ashokka et al. [42, 43]; Canning et al. [36]

##### Patient participation

**Fig. 3:**
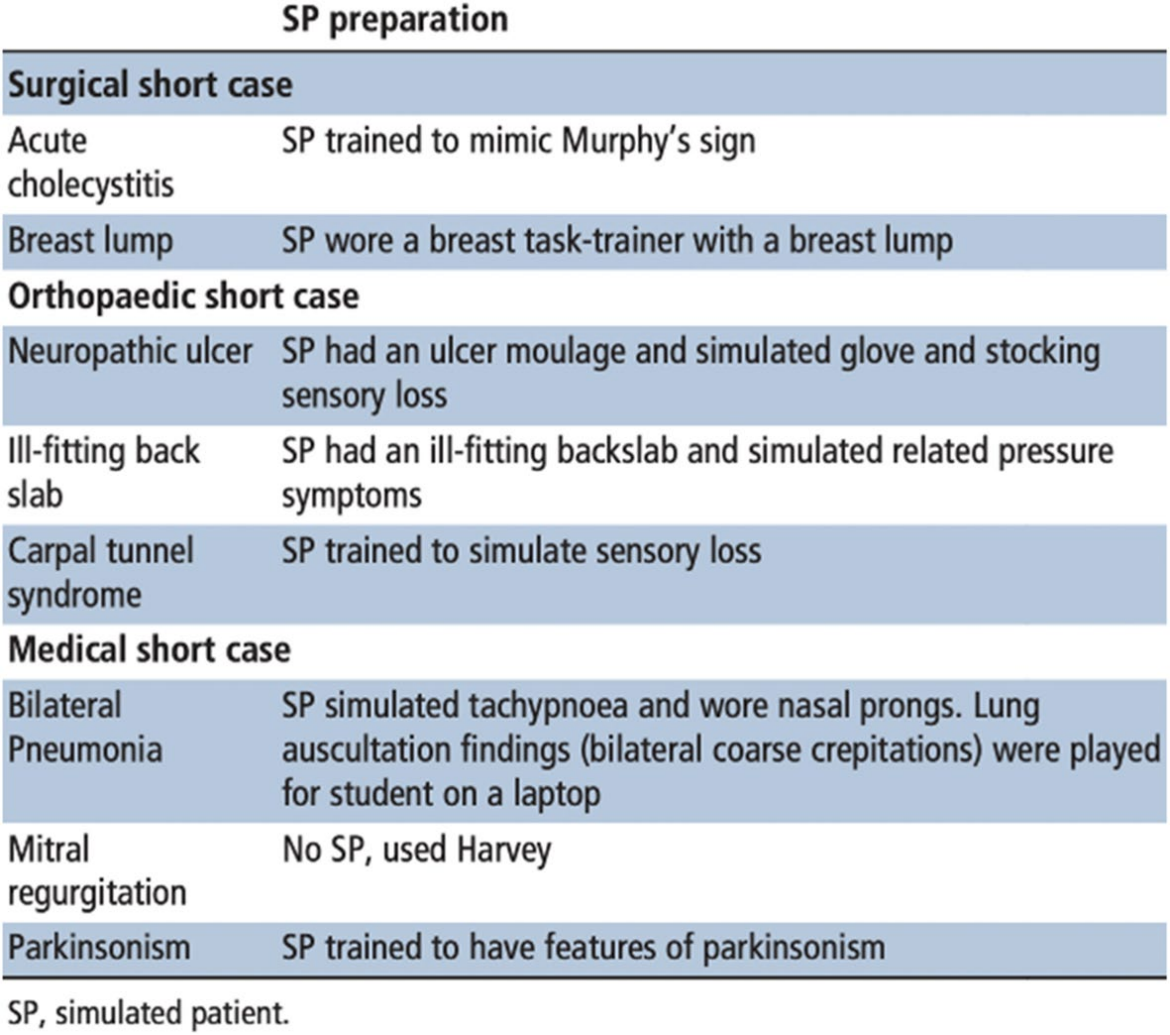
Examples of simulated patient cases at National University, Singapore [73]. (Taken with permission from the study author)

Study authors described modifying their usual approach to patient participation in response to the pandemic. Two studies described replacing patients with mannequins and simulators [38, 45] and five studies described using hybrid stations that mixed a professional encounter with patients/simulated patients and a physical examination/practical procedure demonstrated on a mannikin or task trainer [36, 40, 42, 49]. Several studies explained how they increased the use of simulated patients in stations: one described how simulated patients were trained to mimic clinical conditions by eliciting clinical signs (examples given in Fig. 3 from National University, Singapore) [49, 73] while two others described using visual aids to support simulated patients in their role [44, 45], e.g. make-up artistry and wigs that were used to transform actors into elderly patients [44]. Three studies described how they safely used patients with specific medical conditions, allocating each a nurse to assist them [35, 46, 50].

##### Station content

Two studies described history-taking stations which were undertaken via videoconferencing with patients offsite while students remained onsite [36, 38]. Additionally, two studies described replacing activities used in traditional stations [41, 46]; one study replaced fundoscopic examination with interpretation of retinal photos [46], and another replaced patient encounters with questioning from an examiner [41].

#### Study author evaluations

##### Reported outcomes

Four studies reported pass/fail rates were comparable to those seen in previous years’ cohorts [39, 45, 46, 48, 50]. Four studies reported positive feedback was received from participants and stakeholders regarding the clinical examination [35, 42, 44, 45]; this included participants describing the examination as “smooth and successful” [42] and external examiners commending the defensibility of the examination [35]. No recorded cases of COVID-19 transmission were reported in participants by the five studies that investigated this outcome during their respective follow-up periods [35, 36, 46, 49].

##### Challenges (including recommendations)

Several challenges were described. Some studies reported that participants were anxious about being exposed to COVID-19 [36, 40, 46, 49]; one study noted that some patients declined invitations to participate because of this fear [46]; several authors recommended holding regular briefings and check-ups to reassure participants in future years [45, 49]; and one study recommended offering compensation for patients if they contract COVID-19 after the examination [49]. Some authors also described difficulties in examination planning. Two studies described how their plans were constantly disrupted due to the changing local COVID-19 situation in Singapore such as changing lockdown rules [42, 49]. Additionally, authors described difficulties in recruiting sufficient numbers of clinical staff due to their clinical deployment in the pandemic [35, 46]. The validity of the examination was also described as a challenge; one study reported how the exclusion of severely ill patients meant that the range and severity of clinical conditions could not be fully represented [36, 49]. One study also reported how planned infection control measures incurred a high cost [46].

##### Successes (including recommendations)

Several studies noted that their teamwork and planning had been a success and recommended a significant investment in the planning of future examinations [35, 36, 42, 49]. One study additionally recommended conducting a thorough risk assessment and tailoring risk mitigation strategies accordingly, as well as ensuring there is sufficient PPE available for all attendees [36]. Two studies reported successful use of technology in the examination, using digital scoring and recording student performance as well as using videoconferencing in history-taking stations. These studies recommended use of technology in future examinations [36, 38].

### Online examinations

#### What was done?

##### Circuit structure

**Fig. 4:**
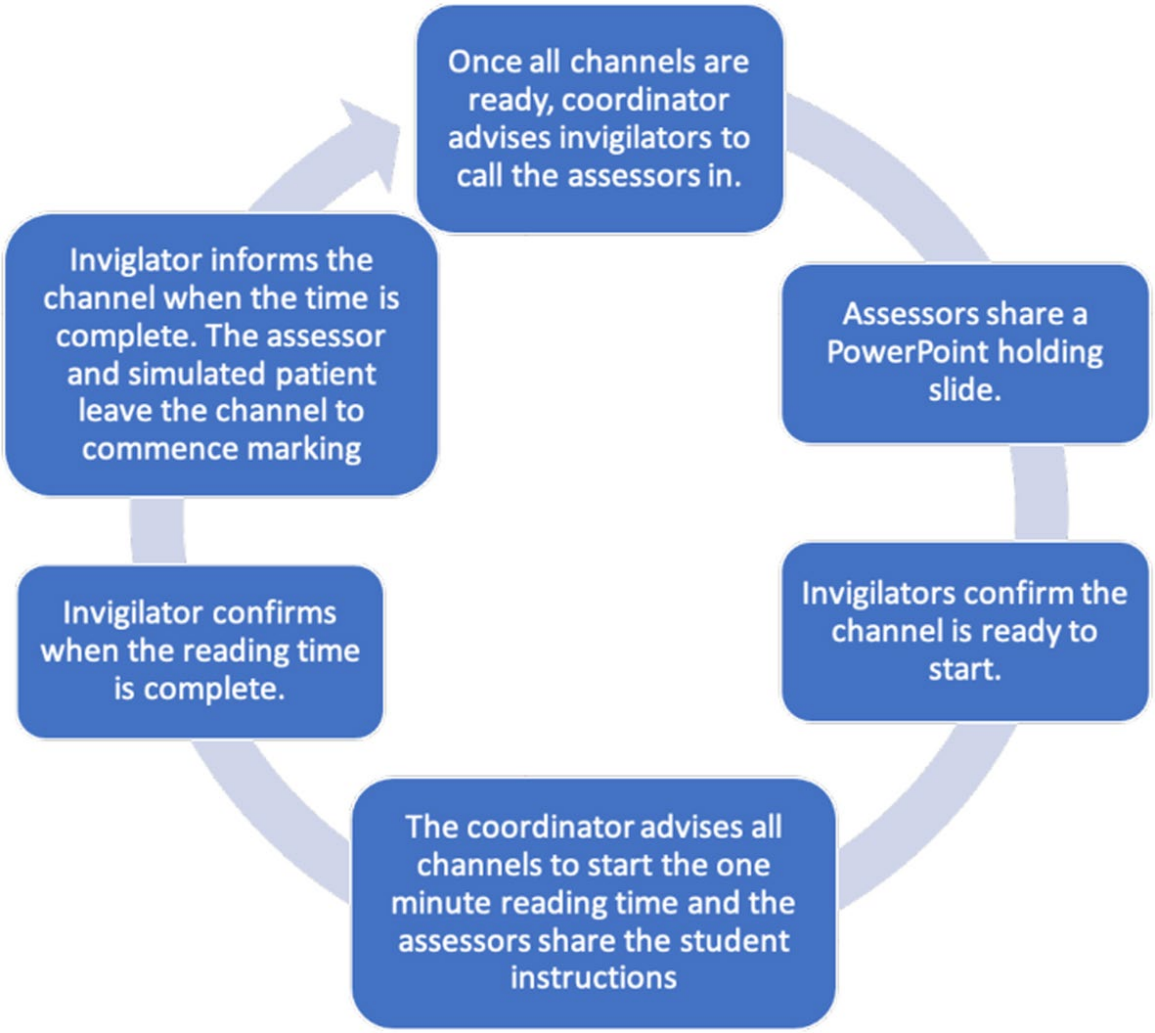
A diagram to illustrate the OSCE cycle on Microsoft Teams at the University of Buckingham [74]. (Taken with permission from the study author)

Authors described attempting to make the online examinations resemble in-person examinations by creating different online rooms as part of a circuit structure using the functionality of videoconferencing software. Several online resources and software were used, as shown in Table 4. In Zoom, this was done using the ‘breakout room’ function [45, 51, 55, 56, 60, 63, 66, 69, 71]; candidates started in a main Zoom room where they were briefed or given pre-encounter notes [62–66], subsequently they joined individual Zoom ‘breakout rooms’ where examiners and patients were pre-positioned. Once the candidate-patient encounters were complete, candidates filled in their post encounter notes and were placed back into the main Zoom room, and the process repeated for the next case [60, 63]. Other studies reported using similar functions in Microsoft Teams [37, 52, 62, 68, 71], where candidates remained in a ‘channel’ while simulated patients and examiners rotated between candidates. (Fig. 4, a visual representation of the OSCE structure on Teams from the University of Buckingham) [52, 71, 74].

**Table 4:** Technological software described and their uses

Use of the software:	Software name:	Studies that used the software:
Hosting software	Zoom (https://zoom.us/)	Anraham et al. [51]; Boyle et al. [53]; Conti et al. [55]; Craig at el. [56]; Farrell et al. [58]; Hannon et al. [61]; Ryan et al. [66]; Setiawan et al. [67]; Shaiba et al. [69]; Stewart et al. [71]
Hosting software	Microsoft Teams (www.microsoft.com/microsoft-teams)	Blythe et al. [52]; Hopwood et al. [62]; Shaban et al. [68]; Shorbagi et al. [37]; Stewart et al. [71]
External communication for hosts and examiners	Microsoft Teams (www.microsoft.com/microsoft-teams)	Ryan et al. [66]
Pre-encounter door notes and standardised patient checklists on	Qualtrics (www.qualtrics.com)	Hannon et al. [61]
Zoom Examiner marking	Qualtrics (www.qualtrics.com)	Ryan et al. [66]
Pre-encounter instructions and post-encounter notes on Zoom	CAELearningSpace (www.caehealthcare.com/learningspace/)	Lara et al. [63]
Examiner marking form	Google Forms (google.com/forms/)	Shorbagi et al. [37]
Candidate post-encounter notes	A Learning Management System (type not specified by authors)	Major et al. [64]
External communication for hosts, and examiners	Whatsapp (www.whatsapp.com)	Blythe et al. [52]; Hopwood et al. [62]; Shaiba et al. [69]; Shehata et al. [70]
Examiner marking form	Excel (www.microsoft.com/microsoft-365/excel)	Blythe et al. [52]; Stewart et al. [71]
Hosting Software	Skype (www.skype.com)	Brown et al. [54]
Hosting Software	Moodle (www.moodle.com)	Gracía-Seoane et al. [59]; Hamdy et al. [60]
Examiner marking form	Speedwell (www.speedwellsoftware.com)	Shaban et al. [68]
Software designed by authors that gives OSCE administrators control of time of entry and exit of participants in stations	OSCE Time Management Dynamic Website (author designed)	Shaban et al. [68]

##### Patient participation

**Fig. 5:**
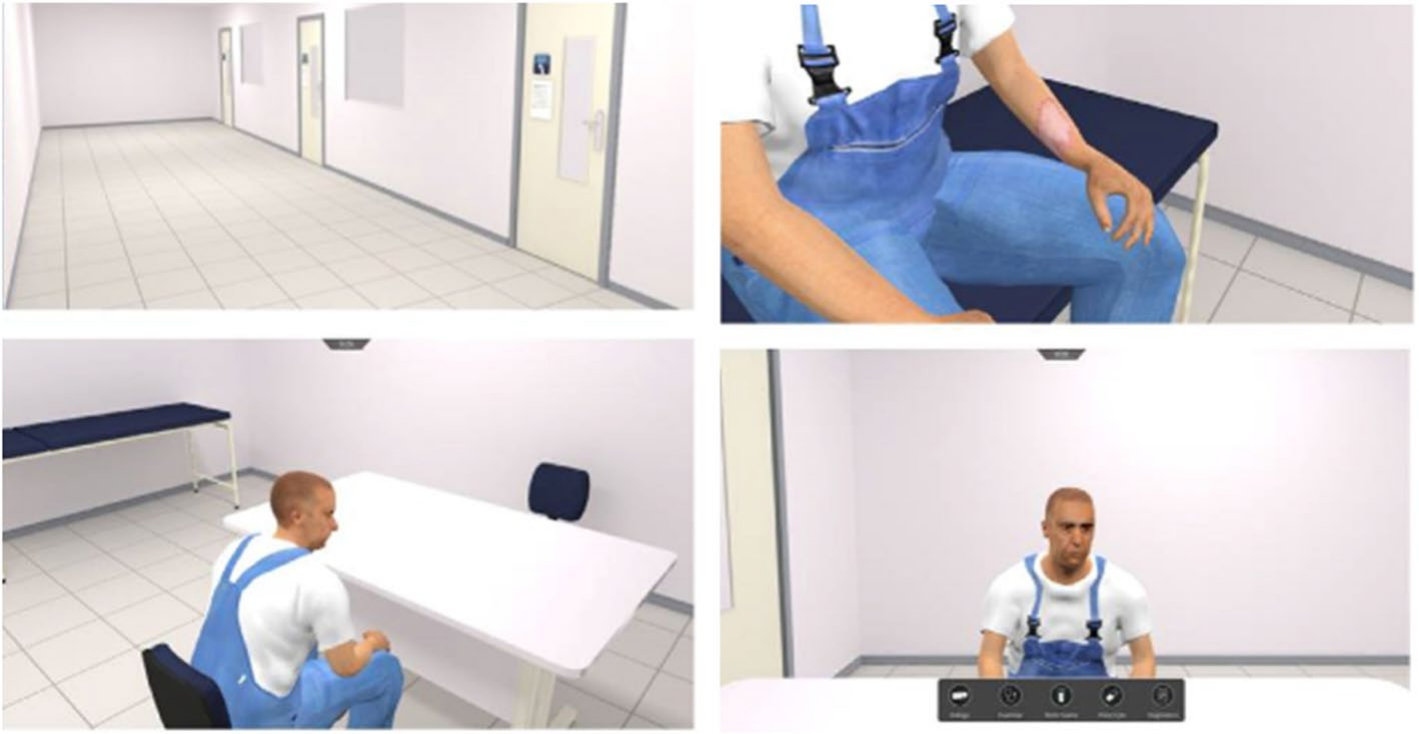
3D prototype of a virtual patient created at Centro Universitario Christus (Campyus Parque Ecologico) [75]. (Taken with permission from the study author)

There was considerable variation between studies in the approach to patient participation in stations. Four studies described holding examinations without real or simulated patients [37, 53, 57, 66]; examiners asked candidates questions based on sequential images or laboratory results shared with the candidate using the ‘share-screen’ function of Zoom [53], or Microsoft Teams [37, 62]. In addition, candidates were questioned on what they would then ask patients in their history to formulate a diagnosis [37, 53]. Two studies incorporated virtual patients [56, 60]; authors in one study created a 3D virtual patient in a consultation room (Fig. 5, from Centro Universitario Christus) [75] using specialist software. Candidates could move 360° around the patient and a virtual script was created with responses activated according to which option the candidate selected, for example, “order exams” with a list of drop-down options to select [56]. Four studies reported the use of standardised patients [55, 56, 63, 69] and seven included simulated patients [52, 62, 64, 66, 68, 71]. Consultations between simulated patients, examiners and students were described as a three-way telehealth consultation with a focus on history-taking skills [66]. Another study described examiners acting as standardised patients [69].

##### Station content

Authors described modifying practical skills and physical examinations, though an explanation of how practical skills were examined was described in only one study [62]. Candidates were sent equipment including suturing equipment, catheters, simulated injections and written documents like notes and drug charts in advance for subsequent use in individual stations, e.g. candidates were asked to suture a banana or vaccinate an orange while on camera during videoconferencing [62]. Three studies described modifications to physical examinations; in two cases candidates narrated physical examinations and verbalised manoeuvres whilst standardised patients verbalised findings [56, 61]. In the other study, candidates were only asked to undertake a neurological examination as the study authors stated it relies heavily on inspection [62]. Two studies described adjusting traditional in-person scoring rubrics to make them suitable for an online clinical examination by modifying physical examination expectations [56, 65], and adding elements of a telemedicine OSCE [65]. One study stated that the new standardised patient checklists, communication scoring tools and faculty observation rubrics would be maintained in the future [63].

#### Study author evaluations

##### Reported outcomes

Overall, study authors reported positive feedback from participants and stakeholders regarding online examinations [37, 54, 56, 58, 60, 62, 66, 68–71]. Eight studies also described how stakeholders greatly valued the training, internet and bandwidth checks, as well as the briefings given to them before the examination [56, 59, 61, 64, 66, 68, 71]. Five studies reported that candidate scores were comparable to those of previous years’ student cohorts [58, 63, 65–68], while one reported that candidate scores were lower compared to previous years [37].

##### Challenges (including recommendations)

A commonly noted limitation of the examinations included the inability to assess physical examination skills [37, 53, 56, 58, 64, 66, 68] and practical procedural skills [37, 52, 53, 68, 71]. Additionally, several studies reported difficulties with internet connectivity. Four studies reported minor technological issues [60, 61, 67, 69]. However, five studies reported no technological issues [53, 57, 62, 68, 71]. Contingency plans for internet connectivity issues included hosting sessions prior to the examination to check the compatibility of stakeholders’ computers with the hosting platform, and ensuring participants had sufficient internet bandwidth [53, 66]. Additionally, several studies reported recording students’ performance for retrospective marking in case internet cut off in the middle of a station and examiners were unable to mark the station in real time [37, 51, 53, 71]. Trial runs of OSCEs were also held and were recommended for future examinations to identify potential problems [37, 52, 62, 65, 66, 68, 71]. Additionally, authors found that using a hosting platform familiar to candidates through previous teaching reduced the chance of technological or compatibility problems arising [51, 62, 66, 71].

##### Successes (including recommendations)

Improvements in examination planning was frequently described in the studies and all recommended taking a more active and detailed approach to planning in the future [52, 62, 68, 70, 71]. Several studies recommended ensuring that adequate numbers of staff (including a ‘super-host’/controller host) should be made available [62], and these staff should have a more diverse set of skills (including IT skills) [37, 70, 71]. Additionally, several studies noted the need to prepare sufficient resources; one recommended distributing documents prior to the examination, including instructions for candidates (contacts for troubleshooting, videoconferencing instructions, and camera and microphone set-up) [70]. Another study recommended continuing to print copies of all relevant documents (e.g. marking grids) so examiners and hosts could concentrate on the candidate on the screen [62]; this study also recommended sending candidates a list of equipment before the examination (e.g., suturing equipment), but that this list not be limited to the specific examination stations, so students cannot predict what will come up. Several studies emphasised the importance of adequate training and communication for all participants [66, 68, 70]; one study noted that ‘over-communication’ is very important, especially for candidates [66]. Three studies recommended using Zoom due to its functionality [54, 70], and in general, study authors reported that examinations had been successful and effective at discriminating between candidates on the basis of the comparability between candidate scores this year and from years’ cohorts [53, 56, 59, 62, 66, 68, 71].

##### Future plans

Five studies indicated future plans to hold online OSCEs [51, 61–63, 67, 71]; two reported imminent plans to continue online OSCEs during the pandemic [61, 66] and five indicated plans to use online OSCEs beyond the pandemic [37, 51, 62, 63, 71], noting that it could be a beneficial tool for students on remote placements [62] or that it saved time and resources compared to in-person examinations [37, 63]. One study reported wanting to return to traditional face-to-face OSCEs when the pandemic permits [53] and two studies noted that while virtual clinical examinations held promise in the pandemic [60, 69], it was important to recognise their limitations, especially in widening the technological deprivation gap between students [69]. Seven studies referred to telehealth in their conclusions, where telehealth is defined as the “delivery of health care services, where patients and providers are separated by distance” [76]; authors noted that online OSCE stations could be a useful tool for examining telehealth skills [56, 62, 63, 65]. The authors who created the 3D prototype patient (Fig. 5) also indicated more research is needed into the functionality of this 3D prototype for examining medical students [56].

## Discussion

### Summary of results

To the best of our knowledge, this systematic review represents the first synthesis of the approaches adopted by Medical Schools to undertake clinical examinations in context of restrictions imposed by the COVID-19 pandemic. Our review found that there were two main approaches to conducting the examinations: adaptations to in-person examination or a switch to online examinations.

Study authors describing in-person clinical examinations recounted deploying stringent infection control measures in conjunction with modifying station content and patient participation to reduce the risk of transmission of COVID-19. Common adaptations included replacing real patients with simulated patients and utilising mannequins or task trainers for practical skills or physical examination skills assessment. None of the studies that recorded postliminary COVID-19 cases reported any cases of transmission in the examination participants, though many study authors reflected that it was a challenge to address participants’ fear of catching COVID-19. Commonly articulated successes and recommendations included good teamwork in the planning of the examinations.

Study authors describing online clinical examinations reported devising OSCE circuits on online software and modifying station content to enable delivery online. Zoom and Microsoft Teams were the most common assessment hosting platforms; online OSCE station rooms were constructed using ‘breakout rooms’ or ‘channels’ accordingly. Studies frequently reported replacing candidate-patient interactions with examiner questioning where candidates would be asked to verbalise physical examinations/manoeuvres in lieu of actually performing the physical examination. Indeed, all study authors noted the inability to assess physical examination and practical procedural skills as a major limitation to online delivery of examinations as well as the heavy dependence on a stable internet connection this approach requires for all participants. Future recommendations included hosting online briefings to check internet bandwidth and computer compatibility prior to the examination (which was also listed as a common success). Study authors also noted that with sufficient planning and development, online examinations could be effective at examining clinical skills and indicated that in future, online clinical examinations could be very useful for examining students on remote placements and provided an authentic approach to assessing telehealth skills.

In both approaches to adapting clinical examinations, candidate scores were reported to be comparable to previous years’ student cohorts and there was generally positive feedback received from participants and stakeholders.

### Quality of the evidence base

Unlike research with outcome measures, most studies included in our review focused on sharing practises, therefore we elected to assess for risk of reporting bias in line with Gordon et al. [12]. It is understandable that amid a pandemic there is demand for practice-developments and research to be disseminated swiftly, which means that authors might not undertake outcome evaluation due to pressures of time and the pandemic response. Nonetheless, it is imperative authors uphold rigour in the reporting of medical education developments. Our review considered that high quality reporting should describe the: reasons for the adaptation of the clinical examination; setting of the examination; resources used; and evaluation in the form of study author reflections or research outcomes. Omission of any of these key details would make the reported adaptation to clinical examinations less reproducible across different contexts. Unfortunately, few papers in our review met these reporting criteria fully our risk of bias assessment highlights the heterogeneity in reporting of medical education developments. Part of the explanation for this could be that several studies were published as short reports. However, this also emphasises the necessity for systematicity in the reporting of medical education developments.

### Comparison with existing literature

Though no prior systematic review has specifically considered the adaptations to clinical examinations required by the pandemic, two systematic reviews examining medical educational developments more broadly have been published; Gordon et al. [12, 13] and Dedeilia et al. [11]. Both reviews included sub-sections on assessment; Dedeilia et al. [11] described teleconferencing to assess clinical skills and Gordon et al. [12] briefly outlined the use of online OSCEs and in-person OSCEs with additional infection control measures. However, neither review provided more than a brief description of the adaptations over a few sentences. Four studies from our review overlapped with those identified by Gordon et al. [12], who undertook their searches in May 2020 whilst ours ran to October 2021; from May 2020 to October 2021, numerous medical education papers were published at a fast rate so we were able to identify and incorporate a number of additional papers in our review. In common with our findings, Gordon et al. [12] also noted that included studies did not report developments in sufficient detail and can be regarded as low quality in terms of reporting bias in the context of medical educational developments. They recommended future study authors use the questions- ‘what?’, ‘so what?’, ‘now what?’ when reporting developments (also known as Borton’s model of reflection) [77].

### Strengths and limitations

The strengths of our review included the systematicity and methodological rigour we employed throughout. We performed comprehensive literature searches including key bibliographic databases, key journals, grey literature and relevant websites. This meant we encompassed a range of sources and retrieved international studies, including those published in languages other than English. Additionally, we found substantial agreement between the two reviewers at both stages of screening. We effectively piloted eligibility criteria and the data extraction form prior to commencing each stage of our review and adapted a pre-existing medical education development reporting risk of bias tool to make it suitable for use in our review. Furthermore, two reviewers independently assessed a sample of studies for risk of bias, in addition to undertaking extensive discussions with senior authors to determine suitable thresholds for quality ratings in each of the four risk of bias assessment domains. Finally, we updated all searches in October 2021 given the currency of our review.

There were some limitations to our study. It was challenging to develop a search strategy that was sufficiently sensitive to capture key papers, yet specific enough to make it feasible to screen the retrieved papers in a timely manner. This may have resulted in missed studies and was compounded by the observation that medical education publications were indexed inconsistently in the databases we searched. The approaches of just 48 Medical Schools were included in our review; however, all Medical Schools internationally would have had to adapt their approaches to clinical examinations in the pandemic. Numerous factors could have influenced whether Medical Schools sought to publish adaptations to their practices, such as local COVID-19 restriction timescales, familiarity with the research and publication process, and staff capacity; however, exploring this is not within the scope of this review. Of the 39 Medical Schools included, a significant proportion of studies originated in Asia, meaning our review may not fully represent the international range of approaches that Medical Schools adopted to facilitate clinical examinations. Finally, our risk of bias assessment was subjective, and occasionally it remained unclear whether studies should be rated low quality, unclear quality or high quality in each of the four domains.

### Future recommendations

Firstly, regardless of the approaches adopted, study authors all concluded that it was feasible to adapt existing clinical examinations and deliver effective assessments despite the restrictions placed on Medical Schools by the pandemic. Commonly stated enabling factors to the success of these adaptations were sufficient workforce and other resources, and the investment in time for planning and carrying out these examinations (which may take longer than traditional clinical examinations), and we would urge that medical educators are given adequate time, facilities, and resources in order to plan and deliver the necessary changes.

Several study authors describing a move to online clinical examinations concluded that these approaches were unable to assess key domains such as physical examination skills and practical procedural skills. Therefore, adaptations to in-person clinical examinations with stringent infection control measures may be preferred where possible. However, online delivery of clinical examinations may provide a useful tool for examining telehealth skills. The advent of ubiquitous internet usage, which has been expedited by the pandemic, means that telehealth has become a prominent mode of delivery in many settings. Telehealth demands a distinctive skill-set compared to conventional face-to-face consultations, in which medical students will need to gain skills and demonstrate competence in the future [78, 79]. Some recent studies describe the development of a telehealth curriculum [80, 81] and we prompt further primary research to establish the optimum approach for telehealth curriculum delivery and examination of telehealth skills of medical students.

In addition, we would also recommend that Medical Schools and regulatory bodies work together to ensure adequate contingency plans are prepared for future disruptions to critical clinical assessments, for example, having a blueprint for an online clinical examination ready for immediate use. Moreover, we urge medical educators to consider whether their assessment strategy is resilient to future disruptions, for example through reducing reliance on final year high-stakes clinical examinations and more focus on programmatic assessment and work-based assessments. Medical educators could consider a model such as Miller’s pyramid; a framework which emphasises the need for different assessment modalities for different expected outcomes in medical education [82]. This enables Medical Schools to draw on a body of evidence regarding the competence and performance of individual students if it were not possible to proceed with planned clinical examinations in the future.

Because of the disparities in both the indexing and reporting of clinical examinations described in the studies included in our review, we recommend that medical education research and publication continues to work towards increased systematicity. Examples of highly systematic work that readers might study include BEME guides [16, 83]. Increased systematicity could include agreed common terminology for medical education research that can be used when indexing studies. Additionally, we would recommend that either Gordon et al.’s [12]. ‘what?’, ‘so what?’, ‘now what?’ questions be considered when reporting developments in medical education, or that a framework for reporting these developments be created afresh. We appreciate that reporting changes in education practises are different to the standard reporting of primary research. However, greater systematicity in reporting will improve the ability to synthesise and disseminate new practises and enhance the reproducibility of study findings across wider contexts.

## Conclusions

We conducted a systematic review to identify the approaches that Medical Schools, internationally, used when conducting clinical examinations of medical students in the COVID-19 pandemic. We identified two broad approaches, adaptation to in-person examinations and a switch to online clinical examinations. Study authors reporting both types of adaptation concluded that conducting clinical examinations was feasible, but it required a significant investment in planning, time, and resource. Of note, a major limitation to online examinations was the inability to examine physical examination skills or practical procedural skills, though other advantages of online approaches are a potential area for further research. We hope our review will be used as a resource by the international medical education community to understand what adaptations have been made in response to the pandemic to help design future clinical examinations in their own institutions that meet local needs; to facilitate reflections on past practises; and to determine what should remain post-pandemic.

## Data Availability

All data generated or analysed during this study are included in this published article [and its supplementary information files].

## Abbreviations

AMEE: An International Association for Medical Education
ASME: The Association for the Study of Medical Education
BEME: Best Evidence Medical Education
COVID-19: Coronavirus Disease
ERIC: Education Resources Information Centre
MeSH: Medical Subject Headings
OSCE: Objective Structured Clinical Examination
PPE: Personal Protective Equipment
PRISMA: Preferred Reporting Items for Systematic Reviews and Meta-Analyses
STORIES: STructured apprOach to the Reporting In healthcare education of Evidence Synthesis
VICEE: Virtual Clinical Encounter Examination
